# Ophthalmic Rosai–Dorfman disease: a multi-centre comprehensive study

**DOI:** 10.1186/s12886-021-02173-1

**Published:** 2021-11-23

**Authors:** Tariq A. Alzahem, Antonio Augusto Cruz, Azza M. Y. Maktabi, Fernando Chahud, Hind Alkatan

**Affiliations:** 1 Vitreoretinal Division, King Khaled Eye Specialist Hospital, Riyadh, Saudi Arabia; 2 Ophthalmology Department, College of Medicine, King Saud University Medical City, King Saud University, Riyadh, Saudi Arabia; 3 Ophthalmology Department, School of Medicine of Ribeirão-Preto, University of São Paulo, São Paulo, Brazil; 4 Pathology and Laboratory Medicine Department, King Khaled Eye Specialist Hospital, Riyadh, Saudi Arabia; 5 Pathology Department, School of Medicine of Ribeirão-Preto, University of São Paulo, São Paulo, Brazil; 6 Department of Ophthalmology, College of Medicine, King Saud University, P.O.Box 266, Riyadh, 11362 Saudi Arabia; 7 Pathology and laboratory Medicine Department, College of Medicine, King Saud University, Riyadh, Saudi Arabia

**Keywords:** Rosai–Dorfman disease, Orbit, Familial, Histopathology, Lymphadenopathy

## Abstract

**Background:**

To provide basic demographic information and clinicopathologic features of ophthalmic Rosai–Dorfman disease (RDD) with a literature review.

**Methods:**

A multi-centre retrospective case series reviewing all patients with histopathologically confirmed ophthalmic RDD at three tertiary eye care centres between January 1993 and December 2018.

**Results:**

Eleven eyes of eight patients with histopathologically confirmed ophthalmic RDD were included, with equal numbers of males and females. The median age was 40.25 years (range: 26.6–72.4). Two patients had familial RDD. The orbit was the most commonly involved site (90.9% eyes). One patient (one eye) presented with a scleral nodule, anterior uveitis and cystoid macular oedema. Visual acuity ranged from 20/25 to light perception. Six patients had an extra-nodal ophthalmic disease, and the remaining two had an associated submandibular lymphadenopathy (nodal RDD).

**Conclusions:**

Ophthalmic RDD can be the only manifestation of this systemic disease, with the orbit being the most commonly involved site, exhibiting bone destruction, intracranial and/or sinus involvement and variable degree of visual loss. Ophthalmic familial RDD represent a severe form with a malignant course. Steroid monotherapy may be inadequate to control orbital RDD; thus, combined treatment is usually necessary. A comprehensive approach to assessment and management is recommended.

## Introduction

Rosai-Dorfman disease (RDD) is a rare histiocytic disorder first described by the French pathologist Destombes in 1965 and then characterized by Juan Rosai and Ronald Dorfman in 1969 [1, 2]. Previously denoted as sinus histiocytosis with massive lymphadenopathy, this name was replaced by RDD due to the variety of extra-nodal disease signs and occasional lack of lymphadenopathy [3]. The word “sinus” in the original term refers to lymph node sinuses rather than paranasal sinuses. As the previous name implies, RDD features massive, painless lymphadenopathy that is characteristically self-limited [2]. The classical RDD description comprises the involvement of lymph nodes in the head and neck areas with cervical lymphadenopathy in over 80% of cases [4]. Therefore, bilateral massive cervical lymphadenopathy is a common presentation. Constitutional symptoms, including fever, night sweats, fatigue and weight loss, commonly occur [2]. The involved lymph nodes may also include mediastinal, inguinal and retroperitoneal nodes [5].

Extra-nodal involvement sites in sporadic RDD have been documented in 43% of cases [6]. The skin, paranasal sinuses, bone and orbital tissues are the most frequent extra-nodal sites [6]. Central nervous system (CNS) involvement usually occurs without extracranial lesions [7]. Ophthalmic manifestations occur in approximately 11% of cases, but isolated ophthalmic disease without lymphadenopathy is extremely rare [4, 8]. The orbit is the most commonly involved site in ophthalmic RDD [8, 9]. RDD can also present as an epibulbar mass, scleritis, uveitis or serous retinal detachments with choroidal involvement [8–10]. Cases with compressive optic neuropathy and lesions mimicking optic nerve and lacrimal tumours have been reported [11].

The histiocytic proliferation has been proven as polyclonal, reactive and non-neoplastic [12]. In the revised classification of histiocytoses and neoplasms of macrophage-dendritic cell lineages, RDD belongs to the R group, which is further classified into five main subgroups, including familial RDD, classical (nodal) RDD, extra-nodal RDD, neoplasia-associated RDD and immune-disease-associated RDD [5, 13].

This article presents the variable clinical manifestations of histopathologically proven ophthalmic RDD cases. Our cohort includes patients with nodal, extra-nodal, sporadic and familial RDD. We also report our experience in the management and outcomes of this rare disease.

## Methods

Eight patients (11 affected eyes) were diagnosed with ophthalmic RDD at the King Khaled Eye Specialist Hospital (KKESH), King Abdulaziz University Hospital (KAUH), Riyadh, Saudi Arabia, and the University Hospital of Ribeirão Preto Medical School (HCFMRP), Ribeirao Preto, Brazil, between January 1993 and December 2018. All diagnoses were established by tissue biopsy performed at the study centres. This multi-centre, retrospective, noncomparative study was approved by the Human Ethics Committee/Institutional Review Board at KKESH with a collaborative agreement among KKESH, KAUH and HCFMRP which adheres to the tenets of the Declaration of Helsinki. Informed written consent was obtained from patients for publication purposes.

From medical records, we extracted demographic data such as patient age at presentation, gender, information on symptoms and their duration, ophthalmic/general examinations, imaging findings, systemic treatment and follow-up. The recorded eye examination findings included visual acuity, laterality, extraocular motility, orbital findings and anterior and posterior segment manifestations. Computed tomography (CT) and magnetic resonance imaging (MRI) results were also documented. Histopathologic findings were reviewed by three pathologists to confirm tissue diagnoses, along with immunohistochemical (IHC) studies whenever performed. Relevant systemic involvement, defined as related systemic disease or multifocal distant lesions with single- or multiple-system involvement including extension, was also extracted. We noted ophthalmic and systemic treatment modalities and management outcomes whenever available.

## Results

Eleven eyes in eight patients with histopathologically confirmed ophthalmic RDD were diagnosed between January 1993 and December 2018 at the study centres. The demographics and clinical data are summarised in Table 1. The gender distribution was equal, and the median age of presentation was 40.25 years (range: 26.6–72.4 years). The youngest two male patients were brothers, indicating a familial form of extra-nodal RDD; however, the parents were not first-degree relatives, and the family history was otherwise unremarkable. Three patients had bilateral disease. The most common presenting complaint was eyelid swelling, reported in 75% of cases. The median symptom duration was 2.25 years (range: 0.16–8 years). The visual acuity of the involved eye at presentation ranged from 20/25 to light perception (LP).

**Table 1:** Demographics and clinical features/intervention of 8 patients (11 eyes) with ophthalmic RDD

Case no.	Age (years)	Gender	Laterality	Visual acuity	Symptoms/signs	Duration of complaints (years)	Relevant Systemic involvement	Orbital localization	Intervention
**1**^**a**^	25–29.9	Male	Bilateral	OD 20/30 OS 20/200	Decreased vision OU, proptosis OU, RAPD OS	8	Bilateral paranasal sinuses infiltration	Intraconal & extraconal in both orbits, secondary optic neuropathy in both eyes	Debulking of masses in both orbits & oral prednisolone, chemotherapy
**2**^**a**^		Male	Bilateral	OD LP OS 20/100	Decreased vision in OD more than OS, left lower eyelid swelling, proptosis OU, RAPD OD	OD 1 year OS 6 years	Isolated brain and facial tumors, bilateral paranasal sinuses infiltration	Intraconal & extraconal in both orbits, right intracanalicular optic nerve compression	Debulking of masse in the left orbit & oral prednisolone, chemotherapy
**3**	30–34.9	Male	Left	20/25	Left lower eyelid swelling, proptosis	1.5	Bilateral submandibular glands enlargement	Diffuse involvement of extraconal and intraconal spaces	Orbital debulking, submandibular gland removal, oral prednisone
**4**		Female	Left	20/25	Left upper and lower eyelid swelling, dystopia, proptosis, inferonasal orbital mass	3	Bilateral maxillary and ethmoidal lesions	Inferior, extraconal	Oral prednisone+ Radiotherapy 4000 cGy
**5**	40’s	Male	Left	20/40	Elevated temporal scleral lesion manifesting as nodular scleritis	0.16	Thickening of the skin of the auricle and subcutaneous nodules of the arm	–	Subconjunctival and oral steroids
**6**	70–74.9	Female	Bilateral	20/100 OU (Bilateral cataract)	Right upper eyelid swelling, enlargement of right lacrimal gland causing dystopia	1	Intracranial/dural lesions	Right supero-temporal and left inferotemporal, extraconal. Enlargement V2 and V3 branches	Oral prednisone
**7**		Female	Right	20/50	Right lower eyelid swelling, proptosis, mild limitation of EOM	6	Bilateral maxillary sinus infiltration and brain extension	Inferior, extraconal	Radiotherapy & oral prednisolone
**8**		Female	Left	20/30	Left upper eyelid swelling, dystopia, ptosis	0.5	Bilateral submandibular glands enlargement	Supero-temporal, extraconal	Oral prednisone

^a^Brothers

*cGy* Centigray, *EOM* Extraocular muscle motility, *LP* Light perception, *OD* Right eye, *OS* left eye, *OU* Both eyes, *RAPD* Relative afferent pupillary defect

The orbit was the primary involvement site in seven patients (10/11 eyes), with one other patient (1 eye) presenting only with a scleral nodule and an associated anterior chamber reaction accompanied by cystoid macular oedema. Six of eight cases had an isolated extra-nodal ophthalmic disease, and the remaining two had an associated submandibular lymphadenopathy (nodal RDD). We further analysed the clinical presentation in these cases according to the involvement site as follows:

### Orbital RDD

**Fig. 1:**
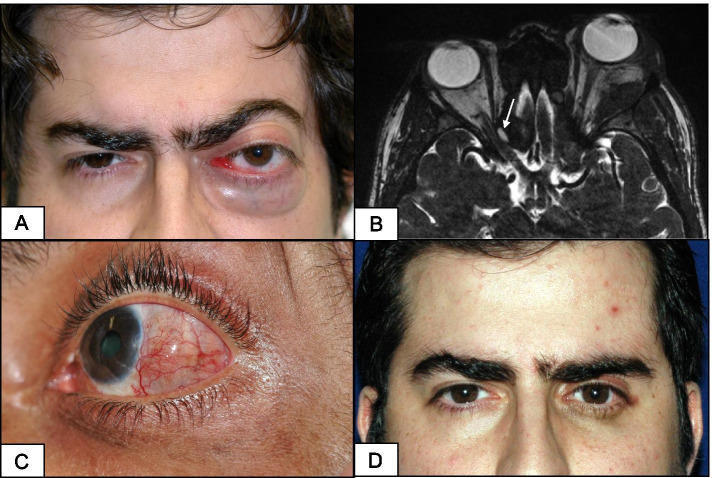
**A** External photo of a male patient (case 3) presenting with a left orbital RDD and a significant left axial proptosis. **B** Axial high-resolution MRI showing bilateral orbital infiltration with right intracanalicular optic nerve encroachment (arrow) in case 2. **C** External photo of a patient (case 5) with scleral RDD showing inflamed and edematous sclera with conjunctival, superficial and deep episcleral vascular congestion. **D** Excellent result was observed in case 3 shown in **A**

Orbital RDD was bilateral in 42.8% of patients. Eyelid swelling and decreased vision were the presenting complaints for 60 and 40% of eyes, respectively. On examination, visual acuity in the affected eyes ranged from 20/25 to LP. A relative afferent pupillary defect was documented in two eyes and mild limitation of ocular motility in one eye. Proptosis and/or dystopia were observed in 9/10 eyes (Fig. 1A). The median proptosis value was 4.5 mm (range: 2–16). Compressive optic neuropathy was seen in three eyes, with an average proptosis of 4.3 mm and no documented ocular motility limitation. A rubbery inferonasal mass was palpable in one patient.

Apart from eyelid swelling and proptosis/dystopia, no other signs of infection or inflammation were observed. Exposure keratopathy secondary to lagophthalmos was documented in one eye. Relevant systemic associations occurred in all patients, including paranasal sinus infiltration (4 cases), intracranial involvement (3 cases), submandibular gland enlargement (2 cases) and facial tumours (1 case). The patients were otherwise healthy. Imaging studies revealed extraconal soft tissue masses in all orbits, with an additional intraconal component in 5/10 orbits. Erosion/destruction of the surrounding bones was observed in all involved orbits. Sclerosis and hyperostosis of the facial bones occurred in one patient. The histiocytic infiltrate involved the paranasal sinuses bilaterally in four patients. Intra-orbital optic nerve compression was documented in two eyes and intracanalicular segment compression in one (Fig. 1B). Intracranial involvement presenting as parenchymal lesions (case 2), extension (case 3) and dural tumours (case 6) was found. Enlargement of the lacrimal gland and V2/V3 branches of the trigeminal nerve was also seen in case 6. Two out of seven patients (cases 3 and 8) were classified as nodal RDD.

### Scleral RDD

A male patient in his late forties (case 5) exhibited an inflamed, elevated, temporal scleral nodule in the left eye (Fig. 1C) for 2 months. The temporal sclera was oedematous and surrounded by a bluish hue, indicating an associated scleral thinning and/or disorganisation of the scleral collagen lamellae. The vascular injection involved the conjunctiva and the superficial and deep episcleral vascular plexuses, which is typical for nodular scleritis. Ultrasound Biomicroscopy of the central nodule showed a thickened ocular wall at the lesion. Moreover, the eye showed a mild anterior chamber reaction and cystoid macular oedema that leaked in fluorescein angiography (not shown). The histiocytic infiltration involved the subcutaneous skin tissue of the left auricle and the subcutaneous tissue of the arm.

### Histopathology and immunohistochemistry

**Fig. 2:**
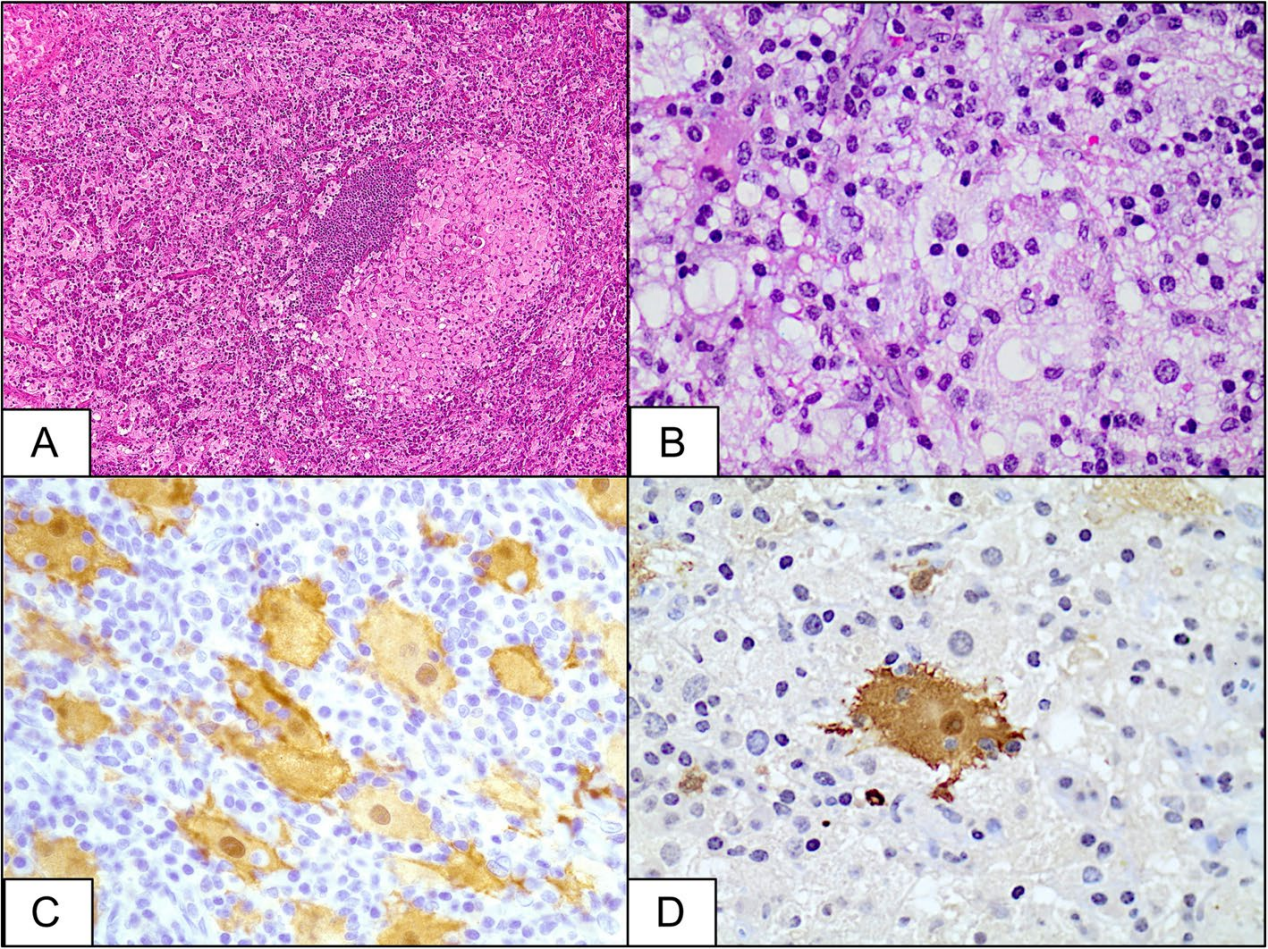
**A** Aggregates of lymphocytic infiltrate surrounded by histiocytes in a patient having orbital RDD (original magnification × 100, Hematoxylin & Eosin). **B** Higher power of the collection of lymphocytes with surrounding large histiocytes (original magnification × 400, Hematoxylin & Eosin). **C** Immunohistochemical staining by S-100 delineating many macrophages engulfing lymphocytes (emperipolesis & lymphocytophagocytosis) for a patient having scleral RDD (original magnification × 400). **D** Another histopathological photo from a patient with orbital RDD showing a typical histiocyte with clear emperipolesis (original magnification × 400 S-100-stain)

The histopathologic sections in the present series had similar characteristics, consisting of soft tissue pieces heavily infiltrated by sheets of histiocytes admixed with lymphocytes, plasma cells and numerous Russell bodies (Fig. 2A and B). Immunohistochemical staining showed that the histiocytes expressed CD68 and S-100, while they did not stain with CD1a. Prominent emperipolesis (lymphocytophagocytosis) was also observed (Fig. 2C and D). We evaluated the IgG4 reactivity in two patients, showing approximately 30 IgG4+ plasma cells/high-power field in one and a non-significant reaction in the other patient.

### Management

All patients with orbital RDD received systemic steroids and exhibited variable improvement in eyelid swelling and proptosis. Two patients received steroid monotherapy (cases 6 and 8), with almost complete resolution of the lesion in case 8, albeit with persistent ptosis, 1 year following the start of a tapering oral steroid regimen. No follow-up data were available for case 6. All other patients received combined therapy, as steroid monotherapy was insufficient to control the orbital disease. Four eyes needed additional debulking procedures due to proptosis severity. This combination of oral steroids and surgical debulking was successful in 1/4 eyes (case 3, Fig. 1D) but failed to stabilise disease progression in 3/4 eyes (cases 1 and 2), with secondary optic neuropathy found at presentation. These cases were referred to oncology service, where chemotherapy was started. The disease thereafter stabilised with no reported progression for more than 2 years. Two patients (cases 8 and 4) received combined oral steroids and radiotherapy; subsequently, the disease stabilised in one patient for 1 year, with no follow-up data for the other patient. The only patient with scleral RDD (case 5) showed complete control with systemic and subconjunctival steroids. The infiltrated auricular skin was the same at the last follow-up visit.

## Discussion

The classic RDD presentation includes massive, bilateral, painless cervical lymphadenopathy. Patients may have fever, fatigue, night sweats, weight loss, arthralgia and pharyngitis [3, 4]. RDD commonly afflicts the head and neck areas, including the orbit and ocular adnexa [14]. Lymph nodes in the mediastinal, axillary and inguinal areas may also be involved [15]. Extra-nodal anatomical locations include the skin and soft tissue (16%), paranasal sinuses (16%), bone (11%), salivary gland (7%), CNS (7%), oral cavity (4%), kidney/genitourinary tract (3%) and respiratory tract/lungs (3%) [4]. Involvement of the kidneys, respiratory tract/lung or liver portends a poor prognosis [4]. In our series, the median age of presentation was 40.25 years, which is comparable to that published by Choi et al. (42 years) but significantly higher than that reported by Vemuganti et al. (13 years) [16, 17]. This might be explained by the slowly progressive disease (median duration of symptoms was 2.25 years in the present series) and that patients waited for several years before seeking medical evaluation. In addition, although the mean age of onset, out of 423 patients published by Foucar et al., was approximately 20 years, RDD in the eighth decade has also been reported [4]. Different races, and thus genetic variability, in our series could also play a role in the onset and natural course of the disease. None of our patients had respiratory or liver involvement.

The orbit and eyelids are the most commonly involved ophthalmic sites, observed in 8.5% of registered RDD cases [4]. Moreover, as the primary manifestation of ophthalmic RDD, orbital involvement can be unilateral or bilateral [8, 17–19]. The lacrimal gland is affected in 8–25% of orbital cases [4, 9]. One in five ophthalmic RDD patients had no evidence of lymphadenopathy [4, 20]. The ratio of associated lymphadenopathy in our patients with orbital RDD was 1:3.5, which is slightly higher than previous reports. The most common extra-nodal involvement sites in ophthalmic RDD patients were the nasal cavity and paranasal sinuses [4, 8], as observed in half of our patients. Intracranial involvement in RDD is rare, although several cases have been reported [21–23]. This involvement may result from direct extension through the orbital apex or may arise as separate brain lesions [18, 19, 24]. These lesions can appear as solitary, extra-axial and homogeneously enhancing dural masses simulating meningioma [25]. Intracranial involvement in the form of extension or isolated lesions was observed in 3/8 patients, suggesting a more aggressive disease in those cases. Older patients and Asians more commonly exhibit cutaneous/eyelid involvement than other patient populations [26]. In the current series, more than 90% of cases had orbital RDD, which agrees with other reports. One patient (case 6) presented with significant lacrimal gland enlargement and concurrent V2/V3 branch enlargement, suggesting a connection to IgG4-related ophthalmic disease (IgG4-ROD); however, this relation was not confirmed histopathologically.

Painless soft tissue mass and proptosis were the predominant reported ophthalmic signs of RDD [8]. This presentation can be severe, as enucleation was needed in one patient with massive orbital infiltration [8]. Other presentations included decreased visual acuity, restricted ocular motility and diplopia, orbital pain, ptosis, dry eyes and eye redness [8]. RDD can also present as epibulbar masses, uveitis, scleritis or serous retinal detachments or may be complicated by compressive optic neuropathy [9–11, 27, 28]. Choroidal masses can also occur, representing intraocular RDD [9]. In this series, proptosis/dystopia was found in 90% of orbital RDD eyes, with a proptosis value reaching 16 mm in one eye along with secondary exposure keratopathy and optic neuropathy. The only patient who presented with an epibulbar scleral nodule had anterior uveitis and cystoid macular oedema.

Inherited conditions can predispose individuals to RDD. Familial RDD has been reported in patients with germ-line mutations in SLC29A3. Such mutations comprise a spectrum of inherited disorders, including familial or Faisalabad histiocytosis, H syndrome and pigmented hypertrichotic dermatosis with insulin-dependent diabetes, which are all described under the umbrella of histiocytosis-lymphadenopathy plus syndrome [29]. Although genetic analysis was not performed for the brothers with orbital RDD (cases 1 and 2) at the time of our study, those patients presented with the most severe and resistant form of RDD. The visual acuity was the worst among all cases; moreover, systemic steroids combined with surgical debulking failed to control the disease, with chemotherapy required to stabilise progression. As reported by Choi et al., younger patients with RDD more often exhibit ophthalmic RDD with poor visual acuity [16]. This trend was observed in our series, with the two younger patients having the worst visual acuity; however, it remains unclear whether this finding is related to the younger presentation or familial pattern.

In general, RDD treatment varies and should be tailored to individual clinical circumstances. Consequently, concepts of first-line and second-line treatments should not be generalised for all patients with RDD. In clinically stable patients, observation is favoured, as the disease usually undergoes spontaneous remission in 20–50% of patients with nodal and cutaneous RDD [30, 31]. Among 80 RDD cases reported between 1969 and 2000, treatment was needed in only half [30]. Therefore, observation may be suitable for patients with uncomplicated lymphadenopathy, asymptomatic cutaneous RDD or possibly asymptomatic extra-nodal RDD. Notably, patients with nodal RDD in this series (cases 3 and 8) showed a generally better response and outcome compared to other extra-nodal RDD patients.

If lesions are isolated and accessible, surgical resection or debulking procedures appear to be the best initial option. Surgery is indicated for lesions affecting organ function or growth and/or quality of life [9, 30]. For progressive and symptomatic orbital masses, surgical excision or debulking is considered standard first-line treatment early in the disease course [17, 27]. Choi et al. reported eight ophthalmic RDD cases, among which four had orbital masses, three had epibulbar lesions, and one had uveitis/scleritis. All patients with orbital RDD received orbitotomy to debulk the masses, with additional chemotherapy in 3/4 patients [16]. Mohadjer et al. reported seven orbital RDD cases with surgical intervention in five cases, performed either initially or during the management course; eventually, these patients experienced recurrence/progression requiring chemotherapy [9].

Steroids may reduce the size of involved lymph nodes and reduce symptoms, but responses are variable. There is no defined optimum dose or duration of use for steroids, but the dose is usually higher than that for other autoimmune diseases [32]. Intralesional 40 mg triamcinolone acetonide improved colour vision and diplopia in a patient with orbital RDD and mild optic nerve compression [33]. However, failure to respond to steroids has also been described [34]. Moreover, relapses are not uncommon after a short period of interruption. It is believed that extra-nodal RDD does not generally show a significant response to steroids [32]. Nevertheless, steroids administered locally or systemically can be practical adjuncts in orbital RDD cases, particularly those with diffuse, residual or recurrent disease [17, 27].

One patient in our cohort exhibited bilateral intraconal RDD that was resistant to multiple combinations of chemotherapies, including prednisone, vinblastine/methotrexate, cyclosporine/ prednisone and vinblastine/celecoxib. Radiotherapy was administered when the patient developed ocular pain with worsening proptosis, followed by disease stabilisation [16]. Another case of progressive orbital RDD that was refractory to surgery and chemotherapy responded well to radiotherapy [27] Standard radiotherapy doses have not been established, but doses of 30–50 Gy have been used [35].

Chemotherapy is usually reserved for refractory or recurrent RDD but may be used as initial therapy in disseminated, life-threatening and organ-threatening diseases. Vision-threatening compressive optic neuropathy and severe/persistent orbital disease are common indications of chemotherapy in ophthalmic RDD [9, 11]. Combining chemotherapeutic agents that suppress B-cell (e.g. rituximab) and T-cell (e.g. methotrexate, cyclosporine) functions has been postulated as the most effective strategy [9]. Oral cyclosporine was successfully used as an adjunct to surgery to limit the long-term side effects of steroids in a patient with perilimbal RDD and sclerouveitis [36]. Chemotherapy with or without steroids was used in 3/4 patients presenting with orbital RDD [16]. In the series published by Mohadjer et al., all patients but one received chemotherapy [9]. Moreover, when genetic analysis identifies *KRAS* activating mutations, that result in the activation of the mitogen-activated protein kinase (MAPK) pathway, MAPK kinase (MEK) inhibitors (e.g. Cobimetinib) can be effective in controlling the disease [37].

In the present series, steroid monotherapy was less likely to control the disease, being sufficient to almost treat the disease in only one orbital RDD case (case 7). When the response to steroids was not satisfactory (cases 1, 2 and 3), surgical debulking was performed, but the following result was excellent in only one eye (case 3). In patients who failed to respond to combined systemic steroid and debulking treatment, chemotherapy halted the disease progression. Another option is radiotherapy, which controlled the disease in one of our patients (case 7). We found that local and systemic steroids were adequate to control epibulbar RDD.

## Conclusions

Ophthalmic RDD is rare and can be the only manifestation of this systemic disease. The orbit is the most commonly involved site, but the ocular surface can also exhibit involvement with intraocular inflammation. The age of presentation varies, with younger presentation and associated family history being risk factors for a poor visual prognosis secondary to compressive optic neuropathy. The absence of an associated lymphadenopathy is common, but the ratio of associated lymphadenopathy in our orbital RDD patients was relatively high. Although reportedly rare, bone erosion/destruction was seen in all of our orbital RDD patients in association with more aggressive disease. Steroid monotherapy may be inadequate to control the orbital disease; thus, combined treatment with surgical debulking, radiotherapy and chemotherapy is usually necessary. Epibulbar RDD can be associated with uveitis and macular oedema, exhibiting a good response to local and systemic steroid treatment. Comprehensive evaluation with a multidisciplinary team is often required.

## Data Availability

The data are available with the corresponding author.
